# The Anticancer Effects of Atractylenolide III Associate With the Downregulation of Jak3/Stat3-Dependent IDO Expression

**DOI:** 10.3389/fphar.2019.01505

**Published:** 2020-01-17

**Authors:** Jun-bao Liu, Dan Chen, Ting-ting Bao, Fang-tian Fan, Chen Yu

**Affiliations:** ^1^Traditional Chinese Medicine Department, People's Hospital of Henan Province, People's Hospital of Zhengzhou University, Zhengzhou, China; ^2^Research Center of Clinical Oncology, The Affiliated Cancer Hospital of Nanjing Medical University & Jiangsu Cancer Hospital & Jiangsu Institute of Cancer Research, Nanjing, China; ^3^Department of Integrated TCM & Western Medicine, Jiangsu Cancer Hospital & Jiangsu Institute of Cancer Research & The Affiliated Cancer Hospital of Nanjing Medical University, Nanjing, China; ^4^College of Pharmacy, Bengbu Medical College, Bengbu, China

**Keywords:** atractylenolide III, indoleamine 2,3-dioxygenase (IDO), interferon-gamma, tryptophan, kynurenine

## Abstract

**Objective:** Indoleamin-2,3-dioxygenase-1 (IDO) has been identified as a checkpoint protein involved in generating the immunosuppressive tumor microenvironment that supports tumor growth. It has been reported that atractylenolide III (ATLIII) has anticancer and immune modulatory effects. This study is to determine the anticancer effects of ATLIII with the Jak3/Stat3-dependent IDO inactivation.

**Methods:** We assessed the cytotoxicity of ATLIII and IFN-γ on lung cancer cells by MTT. We determined the efficacy of ATLIII on IFN-γ-induced IDO expression by RT-PCR and Western blot. We also determined the efficacy of ATLIII on Jak3/Stat3 pathway expression induced by IFN-γ and Jak3/Stat3-dependent IDO activation. Further molecular docking assay predicted the binding activity and site of ATLIII to Jak3 protein. Additional immunofluorescence staining was used to measure the Stat3 intracellular localization. Finally, we performed mouse animal experiments to observe changes in the expression of IDO, p-Jak3, p-Stat3, and tryptophan/kynurenine after ATLIII administration.

**Results:** ATLIII showed no cytotoxicity at a wide of dosage range. ATLIII reduced the phosphorylation level of Jak3 and Stat3 in response to IFN-γ stimulation, then remarkably reduced the nuclear translocation of p-Stat3 by IFN-γ. Lastly, ATLIII significantly downregulated the expression level of IDO at a wide dosage range. Molecular docking assay showed that the oxygen atom on the five-membered ring of ATLIII was capable of forming a hydrogen bond with Leu905-NH2 site of Jak3 protein. Further evidence showed that though IFN-γ had normal capacity to trigger Stat3 phosphorylation, nuclear translocation, and promoter luciferase activity, ATLIII failed to trigger efficacy on reducing these changes under forced Jak3–Leu905 mutant expression condition. Finally, we confirmed this view in *in vivo* experiments.

**Conclusion:** ATLIII has shown significant efficacy to inhibit IFN-γ-triggered Jak3/Stat3 pathway-dependent IDO activation, and do so through a direct binding to Jak3 protein. This study elucidated a new mechanism for the anticancer effect of ATLIII, which may provide a feasible target for the clinical immunotherapy of malignant tumors.

## Introduction

Immunotherapy has shown significance as a novel targeted approach treating cancer by harnessing the body's immune system to fight tumor cells (Pardollar, 2012). T cells have been designed to specifically target tumor cells, which can directly inhibit and kill tumor cells by identifying and aggregating to tumor antigen expression sites and producing long-term immune response (Francisco Rancisco et al., 2009). T cells recognize tumor cells by binding T cell receptor to a major histocompatibility complex (MHC) with specific antigens on the surface of tumor cells (Scott et al., 2010). This interaction is controlled by a series of immunological checkpoints, including co-stimulatory signal and co-suppression signal, which can activate or inhibit T cells (Pratama et al., 2014).

Clinical studies have proven that immune checkpoint inhibitors can reactivate the immune response of T cells by inhibiting the activity of immune checkpoints and releasing the “immune brake” in the tumor microenvironment to achieve an antitumor effect. Immune checkpoint inhibitors have been effective in the treatment of melanoma, lung cancer, gastric cancer, and liver cancer (McNutt, 2013). Passive immunotherapy currently used in the clinical setting is to enhance existing antitumor responses including the use of monoclonal antibodies, lymphocytes, and cytokines, while the small-molecule inhibitors have gradually become a promising research field in cancer immunotherapy (Spranger et al., 2014). Indoleamine 2,3-dioxygenase (IDO) plays an immunoregulatory role associated with tryptophan metabolism (Vécsei et al., 2013). Recent findings reveal that IDO can be triggered by attempted T cell activation, either spontaneous or due to immunotherapy. IDO is only a rate-limiting enzyme for oxidative cleavage of l-tryptophan through the kynurenine pathway and is potentially an attractive target for small-molecule drug development in antitumor immunotherapy (Munn and Mellor, 2007). IDO can be induced in the tumor microenvironment by the spontaneous inflammation and T cell activation associated with many tumors (Munn and Mellor, 2016). IDO has been shown to suppress T-lymphocyte-mediated graft rejection (Jasperson et al., 2008; Godin-Ethier et al., 2011). IDO can also cause T cell suppression after neoplastic transformation by acting as a checkpoint molecule or combining other immune checkpoints, such as cytotoxic T lymphocyte antigen-4 (CTLA-4) and programmed cell death-1 (PD-1), thus preventing the immune system from mounting an effective attack against the cancer (Munn and Mellor, 2013). Therefore, IDO inhibition has potential to become a new type of tumor treatment strategy. Interferons have been shown to be potential anticancer agents. In particular, interferon-γ (IFN-γ) triggers IDO activity and pro-inflammatory cytokine release as distinct cellular programs for endowing allogeneic T cells with regulatory activity (Jurgens and Hainz, 2009). IDO is actually expressed at a low level under normal conditions, and its expression is significantly increased during IFN-γ stimulation in many tumor cell lines (Johnson and Munn, 2012). IFN-γ is known to activate the transcription of IDO *via* the Jak3/Stat3 signaling pathway (Muller et al., 2008; Holtzhausen et al., 2014). In general, IFN-γ binds to its specific receptors IFN-γR1 and IFN-γR2 and causes dimerization of the receptor molecule, thus phosphorylates Jak kinases. The two Jak molecules form a channel to recruit a Stat3 homologous dimer. The Stat3 dimer is then phosphorylated by Jak3, and the phosphorylated Stat3 dimer detach from the receptor. The Stat3 dimer enters the nucleus and binds to the GAS regulatory sequence of IFN-γ-induced gene (Mamane et al., 1999), which indirectly activates the expression of IDO. Previous studies have confirmed that IFN-γ upregulates the expression of IDO on tumor cell membranes through the Jak/Stat signaling pathway (Lee et al., 2006; Wolfle et al., 2011).

Currently, lung cancer has the highest incidence of cancers worldwide. It has been found that the occurrence and the development of lung cancer are closely related to immune tolerance in the local immune microenvironment (Fionda et al., 2013). Atractylodes macrocephala is the rhizome of perennial herb *Atractylodes chinensis*, which is widely used in the treatment of spleen deficiency syndrome (Cai et al., 2012). It mainly contains volatile oils and lactones, such as atractylone, atractyl alcohol, atractylode (ATL), atractylenolide I (ATLI), atractylenolide II (ATLII), atractylenolide III (ATLIII), and atractylenolide IV (ATLIV) (Wang et al., 2009). Some reports showed that volatile oil is the main active anticancer ingredient, while atractylodes macrocephala can improve immune response. Rhizome macrocephala lactones can invigorate spleen and tonifying Qi. Atractylenolide III is a major active product of atractylodes macrocephala. It is not known whether it plays an anticancer role by regulating the immune microenvironment in lung cancer cells.

## Materials and Methods

### MTT Assay

LLC cells were digested with 0.25% trypsin. The digestion was terminated with the addition of a solution containing 10% phosphate-buffered saline (PBS). The cell suspension was mixed and the cell density was adjusted to 1 × 10^4^/ml. The cell suspension was added to a 96-well plate at 180 μl/well for 24 h, and then 2, 4, 8, 16, 32, 64, 128, 256, and 512 μmol/l ATLIII were added to three wells respectively and cultured for 24 h or incubated with 1.875, 3.75, 6.25, 12.5, 25, 50, 100, 200, or 400 ng/ml IFN-γ for 12 h, or firstly incubated with 100 ng/ml IFN-γ for 12 h, then adding 8, 16, or 32 μmol/l ATLIII for 24 h. Fifteen microliters 3-(4,5-dimethylthiazol-2-yl)-2,5-diphenyltetrazolium bromide (MTT) was added to each well and then incubated for 4 h at 37°C in a 5% CO_2_ incubator. ATLIII were obtained from Yong Jian Pharmaceutical Technology Co., Ltd. (Taizhou, Jiangsu, China; purities >98%). DMSO (100 μl) was added to each well. The absorbance was measured at a wavelength of 490 nm using a microplate reader. Cell viability = (OD value of the drug group/OD value of the blank group) × 100%.

### RT-PCR

Trizol was used to extract the total RNA for reverse transcription, and 2 μl of Trizol was taken for PCR amplification. The internal reference was GAPDH; primer sequences for GAPDH: sense: 5′-GTATAATGAGAAGCCAGACCAT-3′, antisense: 5′-ACAGCTTCTCAAGTCT-3′; primer sequences for IDO: sense: 5′-GC AAATGCAAGAACGGGACACT-3′, antisense: 5′-TCAGGGAGACCAGAGCTT TCACAC-3′. The cycling conditions for IDO and the internal reference were as follows: pre-denaturation at 94°C for 2 min, one cycle, denaturation at 94°C for 30 s, annealing at 55°C for 30 s, extension at 72°C for 2 min, for a total of 35 cycles, total extension at 72°C for 6 min. Five microliters of the PCR amplification product was subjected to agarose gel electrophoresis. The electrophoresis bands were observed under an ultraviolet projector and the gray values of the IDO and reference genes were analyzed.

### Western Blot Assay

Lung cancer cells LLC, H1703, H520, PC-9, A549, and H1299 were lysed and the supernatant was collected. Protein loading buffer was added and the mixture was boiled at 95°C for 5 min, followed by electrophoresis in a 10% sodium dodecyl sulfate polyacrylamide gel electrophoresis (SDS-PAGE), transfer to a polyvinylidene fluoride (PVDF) membrane, then blocking with 5% skimmed milk for 1 h. After overnight incubation at 4°C, primary antibodies (β-actin, IDO, Jak3, p-Jak3, Stat3, p-Stat3) were added. Then they were divided into blank control group, 100 ng/ml IFN-γ group, and 100 ng/ml IFN-γ combined with 8, 16, or 32 μmol/l ATLIII group. Labeled goat anti-rabbit IgG (5:1,000) was added after washing with Tris-buffered saline and Tween 20 (TBST) and the blot was incubated at 37°C for 1 h. After washing and incubation for 1 h at room temperature, e*lectrochemiluminescence (*ECL) fluorescence was developed and the bands were exposed to light.

Lung cancer cells were lysed after transfection with the Jak3 protein–Leu905 mutant plasmid, and the supernatant was added to the protein buffer at 95°C for 5 min. It was transferred onto a PVDF membrane after electrophoresis in a 10% SDS-PAGE gel and then blocked with 5% skimmed milk for 1 h. Primary antibodies (β-actin, IDO, Jak3, p-Jak3, Stat3, and p-Stat3) were added overnight at 4°C and the blots were divided into blank control group, 100 ng/ml IFN-γ group, and 100 ng/ml IFN-γ combined with 8, 16, or 32 μmol/l ATLIII groups. After washing with TBST, horseradish peroxidase (HRP)-labeled goat anti-rabbit IgG (5:1,000) was added, reacted at 37°C for 1 h, and incubated at room temperature for 1 h. After washing the membrane, ECL fluorescence was developed and the bands were exposed to light.

### Immunofluorescence Assay

Lung cancer cells were injected into 1 × 10^5^/ml afferent containing glass slides. After being cultured overnight, the cells were washed three times with cold PBS, fixed for 30 min with 4% paraformaldehyde, and finally blocked with goat serum for 30 min. Lung cancer cells received either 100 ng/ml IFN-γ, 100 ng/ml IFN-γ combined with 32 μmol/l ATLIII group, or a non-administration control, then lung cancer cells were incubated by the primary antibody Stat3 (1:200) dilution at room temperature for 1 h. They were then washed with fluorescein isothiocyanate (FITC)-labeled sheep anti-mouse IgG (1:500), incubated for 1 h, washed three times with PBS, and restained for 10 min with 100 ng/ml DAPI. Confocal microscopy was used to observe and photograph the cells.

Lung cancer cells with Jak3 binding Leu905 mutant plasmid were injected with 1 × 10^5^/ml afferent with glass slides. After overnight incubation, the cells were washed three times with ice-cold PBS, fixed with 4% paraformaldehyde for 30 min, blocked with goat serum for 30 min, and 32 μmol/l ATLIII was added. They were then incubated for 1 h at room temperature with Stat3 antibody (1:200), washed with PBS, and then incubated with FITC-labeled goat anti-mouse IgG (1:500) for 1 h. The antibody was removed by washing three times with PBS, and the cells were counterstained with 100 ng/ml DAPI for 10 min and observed under a laser confocal microscope.

### Promoter Luciferase Assay

After being cultured overnight to 70% confluence, Stat3 Wt/Mut plasmid was transfected into lung cancer cells and lysed 24 h after transfection. Then Stat3 Wt/Mut cells were exposed to 8, 16, or 32 μmol/l ATLIII, 100 ng/ml IFN-γ, or a blank control. A 10-μl sample of lung cancer cells was added to 100 μl luciferase detection reagent. Finally, the fluorescence value was measured and the statistical results were calculated.

### CHIP Assay

Lung cancer cells were cultured for 24 h in a 1%O_2_ environment and the nuclear chromatin was then extracted and treated with protein G agarose gel beads. The treated chromatin was then exposed to 5 μg of Stat3 antibody, GAS antibody (as a positive control antibody), or normal mouse IgG (as a negative control) and incubated overnight at 4°C. Using Input DNA as a positive, the effect of chromatin cleavage was verified, and the efficiency of the chromatin immunoprecipitation (CHIP) experiments was calculated according to the sampling ratio in the input. The DNA obtained from the treatment was used for PCR. The PCR primer was the specific sequence of gene promoter region. Each region was amplified separately. The PCR amplification system was 20 μl, including 5 μmol/l primer, 0.25 mmol/l dNTPs, 1 U KOD DNA polymerase, 5% DMSO, and 2 μl DNA template. Amplification conditions were as follows: Firstly, 5 min at 97°C for pre-denaturation, followed by the addition of KOD DNA polymerase for 30 s at 97°C, 30 s at 68°C, 2 min at 72°C, 30 cycles; 30 s at 97°C, 30 s at 59°C, 2 min at 72°C, 10 cycles; and a final extension at 72°C for 10 min. Agarose gel electrophoresis was used to observe the amplification of specific bands and determine the binding of Stat3 to the promoter region GAS of IDO.

### Molecular Docking

The molecular docking experiment of the PBD domain (Jak3 protein) with ATLIII, Jak3 inhibitor 19R, and Jak3 inhibitor 7KV was performed using the AutoDock software for molecular docking. The PDB (4H71) file of Jak3 was downloaded from the Protein Data Bank (PDB), the protein was pretreated, dehydrated, hydrogenated, and processed, and then the active site was determined. ATLIII, Jak3 inhibitor 19R, and Jak3 inhibitor 7KV were then docked with the active site of Jak3.

### Site-Directed Mutagenesis

The Jak3 gene of lung cancer cells was knocked out through CRISPR-Cas9 method, and then the plasmid of a Jak3 mutant leucine 905 was introduced. The sequences of the mutated primers were as follows: wild-type: 5′-GCCTGC AAGA AACG AAAC TCAACCGAAAGCC-3′; leucine 905 mutant: 5′-GCCTGCAAGAAAAGAAACTCA ACCGAAAGCC-3′. The Jak3 mutated primer was designed and synthesized by plasmid and amplified by PCR. The PCR product was digested with 1 μl *Dpn*I restriction enzyme and transformed into the competent cells. The appropriate volume of the transformation system was added to an agar plate containing the antibiotic. The cells were plated onto LB/ampicillin agar plate containing 80 μg/ml X-gal and 20 mmol/l isopropyl β-d-1-thiogalactopyranoside (IPTG) and the plates were incubated. The clones formed by the transformation of the mutant reaction system were blue clones on the agar plate containing X-gal and IPTG. The mutant plasmids were sequenced, amplified, and extracted for cell transfection. At the same time, the Jak3 protein containing double-stranded oligonucleotide probe was also subjected to the mutant plasmid at the same position.

### *In Vivo* Study

Male C57BU6 mice were purchased from Nanjing Biomedical Research Institute of Nanjing University (Nanjing, China) and were maintained in a HEPA-filtered environment at a standard condition. Transfected LLC cells (1 × 10^7^) were injected in the flank of the mice. After 2 weeks, the subcutaneous xenograft of mice became noticeable. Then the mice were divided into three groups (IFN-γ drug group, IFN-γ plus 100 mg/kg ATLIII drug group, and blank control group). One hundred milligrams per kilogram ATLIII was administered by gavage. IFN-γ (20,000 IU) was administered by subcutaneous injection. Tumor size was measured every 3 days. Three weeks after administration, all mice were sacrificed. High-performance liquid chromatography (HPLC) was used to detect the Kyn and Trp concentrations in peripheral blood by collecting blood from mouse eyeballs. The Kyn/Trp ratio was used to assess IDO1 activity. The tumors were excised and weighed. Tumor volume was determined with the formula (*L* × *W*2)/2, where *W* indicates the perpendicular minor dimension whereas *L* represents the major one, respectively. The tumor tissues were collected for H&E staining analysis of p-Jak3 and p-Stat3.

## Statistical Analysis

All data are expressed as the mean ± SD, and each experiment was repeated three times. All data were analyzed in the SPSS 19.0 statistical software (SPSS Inc., Chicago, IL, USA). Statistical differences were compared by *t* test or one-way ANOVA. *p* < 0.05 indicates that the difference was statistically significant.

## Results

### Assessment of the Cytotoxicity of ATLIII by MTT Assay

**Figure 1:**
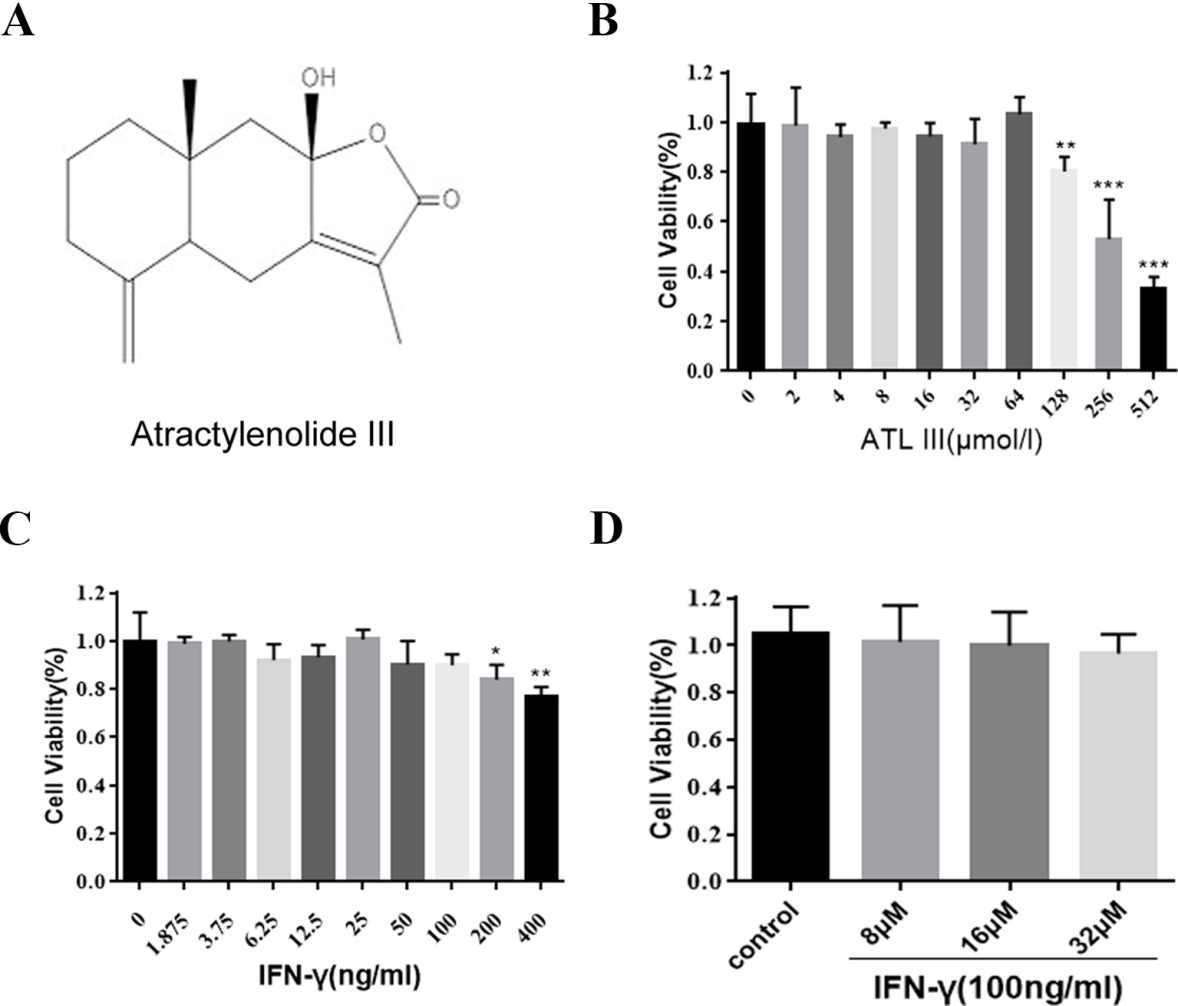
Atractylenolide III (ATLIII) along with IFN-γ did not show cytotoxicity on LLC lung cancer cells. **(A)** Chemical structure of atractylodes macrocephala III. **(B)** Cell inhibition ratios at different concentrations of ATLIII in LLC lung cancer cells. The cell inhibition ratio was determined by the MTT assay. **(C)** The proliferation of LLC lung cancer cells was determined through MTT assay following different concentrations of IFN-γ stimulation. **(D)** The proliferation of LLC lung cancer cells was determined through MTT assay following first 100 ng/ml IFN-γ stimulation, then adding 8, 16, or 32 μmol/l ATLIII for 24 h. The data are representative of at least three independent experiments (mean ± standard deviation). *p < 0.05, **p < 0.01, ***p < 0.001, compared with the control group.

Atractylodes are composed of sesquiterpene lactone compounds. Atractylenolide III (ATLIII) is one of the major ingredients in the atractylodes macrocephala, which is widely used in traditional Chinese medicine (**Figure 1A**). The MTT assay was used to assess the cell toxicity of ATLIII to lung cancer cells. As shown in **Figure 1B**, the wide dosages (2–512 μmol/l) of ATLIII were tested and showed no significant inhibitory effect compared to the control. IFN-γ treatment showed no significant effects on cell viability at the dosage range of 1.875–400 ng/ml (**Figure 1C**). The further combination of IFN-γ at 100 ng/ml with multiple dosages of ATLIII showed no cytotoxicity by the measurement of cell viability (**Figure 1D**).

### IFN-γ Upregulates IDO Gene Expression

**Figure 2:**
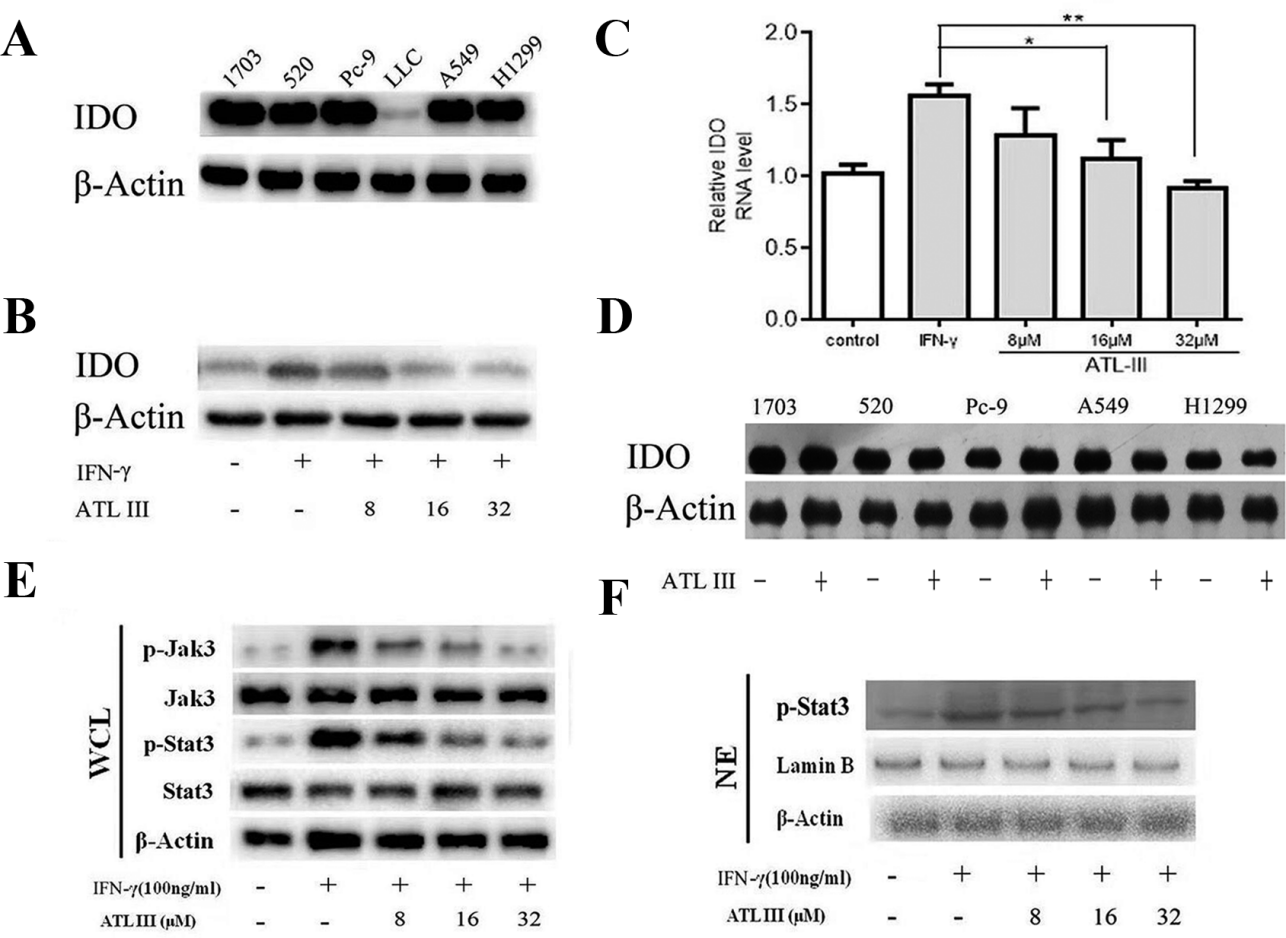
Atractylenolide III (ATLIII) downregulates the expression of indoleamin-2,3-dioxygenase-1 (IDO), p-Jak3, and p-Stat3 induced by IFN-γ stimulation. **(A)** Western blotting was used to detect IDO in six lung cancer cells. **(B)** Western blotting was used to detect IDO in LLC lung cancer cells treated without (or with) IFN-γ (100 ng/ml) and 8, 16, or 32 μmol/l ATLIII. β-actin was used as an internal control. **(C)** RT-PCR detection of IDO expression in LLC lung cancer cells treated with IFN-γ (100 ng/ml) or ATLIII (8, 16, or 32 μmol/l). U6 was used as an internal control. **(D)** Western blotting was used to detect IDO in H1703, H520, PC-9, A549, and H1299 lung cancer cells treated without (or with) ATLIII (32 μmol/l). β-actin was used as an internal control. **(E)** Western blotting was used to detect Jak3, p-Jak3, Stat3, and p-Stat3 in LLC lung cancer cells treated without (or with) IFN-γ (100 ng/ml) and 8, 16, or 32 μmol/l ATLIII. β-actin was used as an internal control. **(F)** Western blotting was used to detect p-Stat3 in the LLC cell nucleus treated without (or with) IFN-γ (100 ng/ml) and 8, 16, or 32 μmol/l ATLIII. β-actin was used as an internal control. The data represent the average of three independent experiments (mean ± standard deviation). *p < 0.05, **p < 0.01 compared with the control group.

We firstly measured the basal levels of IDO protein expression in different lung cancer cells by Western blot. The protein expression levels of IDO showed diversity from different cell lines. IDO protein level was comparable among five cell lines in 1703, 520, PC-9, A549, and H1299 cells and remarkably less in LCC cells (**Figure 2A**). IFN-γ treatment significantly upregulated IDO protein expression levels in LLC lung cancer cells (**Figure 2B**). The similar indication of mRNA levels was shown by RT-PCR (**Figure 2C**). The results confirmed that IFN-γ upregulates IDO activity in LLC lung cancer cells.

### ATLIII Downregulates the Expression of IDO Induced by IFN-γ

In order to test the efficacy of ATLIII on regulation of IDO gene expression, lung cancer cells were pretreated with 100 ng/ml IFN-γ for 12 h following ATLIII treatment at the dosages of 8, 16, and 32 μmol/l for 24 h. As shown in **Figures 2B, C**, IFN-γ stimulation significantly elevated the IDO protein expression levels in LLC cells. Interestingly, ATLIII treatment showed significant downregulation of IFN-γ-elevated IDO gene expression in a dose-dependent manner (8–32 μmol/l). ATLIII at the dosage of 32 μmol/l completely inhibited the IDO protein expression (**Figures 2B, C**). However, we found that ATLIII could not inhibit the expression of IDO protein in 1703, 520, PC-9, A549, and H1299 cells with high expression of IDO (**Figure 2D**).

### ATLIII Inhibits IFN-γ-Induced Phosphorylation of Jak3 and Stat3

Stat3 is the major transcription factor in the IFN-γ-induced signaling pathway. When IFN-γ binds to its receptor, the intracellular region of the receptor is opened. Tyrosine kinase Jak is activated, and then Jak is autophosphorylated. Two Jak form a homodimer groove to recruit Stat3, and the activated Stat3 forms a homodimer for transport into the nucleus, where the homodimer directly binds to the IDO promoter sequence GAS. GAS initiates the transcriptional activity of IDO protein on the tumor cell membrane. To further analyze the molecular mechanism by which ATLIII inhibited the expression of IDO, the cells were pretreated with 100 ng/ml IFN-γ for 12 h following ATLIII treatment at different dosages of 8, 16, and 32 μmol/l. The results showed that the phosphorylation levels of Jak3 and Stat3 were induced by IFN-γ in lung cancer cells (**Figures 2E, F**). ATLIII treatment significantly reduced the IFN-γ-induced phosphorylation of Jak3 and Stat3 with minor effects on their protein expression levels. IFN-γ stimulation also elevated Stat3 protein level in the nucleus, and this was blocked by ATLIII treatment. The results indicate that ATLIII downregulates both Jak3 and Stat3 activation induced by IFN-γ (**Figures 2E, F**).

### ATLIII Inhibits Stat3 Nuclear Translocation

**Figure 3:**
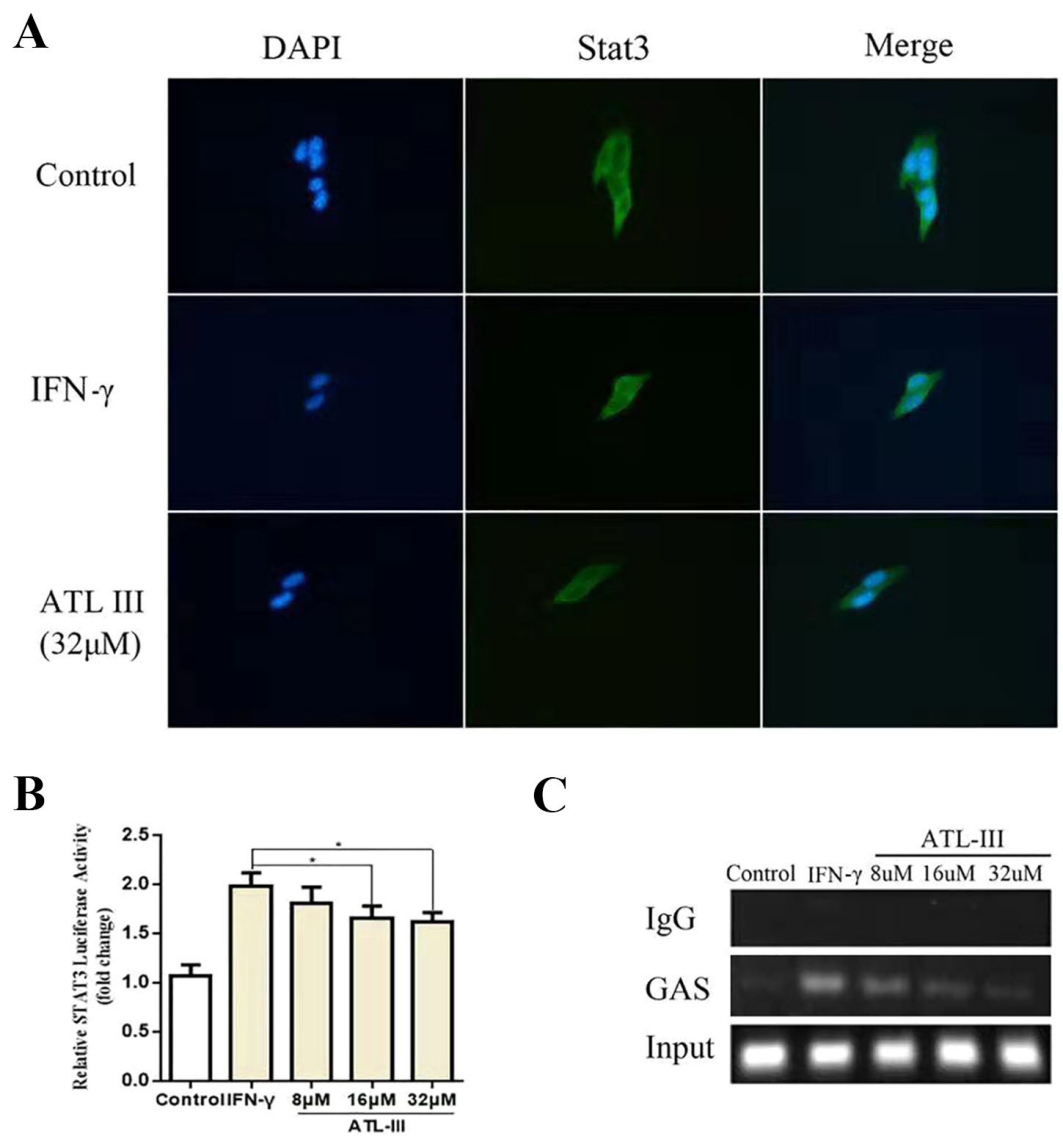
Atractylenolide III (ATLIII) inhibits Stat3 nuclear translocation and its transcriptional activity. **(A)** Immunofluorescence staining detects Stat3 nuclear translocation. **(B)** Luciferase assay detects the transcriptional activity of Stat3. The data represent the average of three independent experiments (mean ± standard deviation). *p < 0.05 compared with the control group. **(C)** Chromatin immunoprecipitation (CHIP) assay was used to detect binding of the Stat3 and GAS promoters.

To further elucidate whether ATLIII inhibits Stat3 phosphorylation, leading to reduced protein nuclear translocation, LLC cells were subjected to immunofluorescence staining and observed by confocal laser microscope. The results showed that Stat3 stains were distributed throughout the cell in the control group. In contrast, IFN-γ treatment resulted in the Stat3 protein localization from cytoplasm to nucleus in 12 h. ATLIII significantly reduced the nuclear localization of Stat3 in the nucleus of LLC cells (**Figure 3A**).

### ATLIII Reduces the Binding Activity of Stat3 to the IDO Promoter Regions GAS

Chromatin immunoprecipitation assay confirmed that the binding activity of Stat3 protein to the IDO promoter regions GAS was enhanced after IFN-γ stimulation, and the binding activity was significantly reduced after treatment with ATLIII in a dose-dependent manner. The dose of 32 μmol/l ATLIII completely abolished IFN-γ-induced binding of Stat3 and GAS (**Figure 3C**).

### ATLIII Inhibits the Transcriptional Activity of Stat3

A luciferase reporter gene assay showed that the transcriptional activity of Stat3 increased after IFN-γ stimulation, then the transcriptional activity reduced after the ATLIII treatment. The transcriptional activity decreased significantly with 32 μmol/l ATLIII (**Figure 3B**).

### Molecular Docking Assay Predicts ATLIII Binding Closely to the Leu905 Site of Jak3 Protein

**Figure 4:**
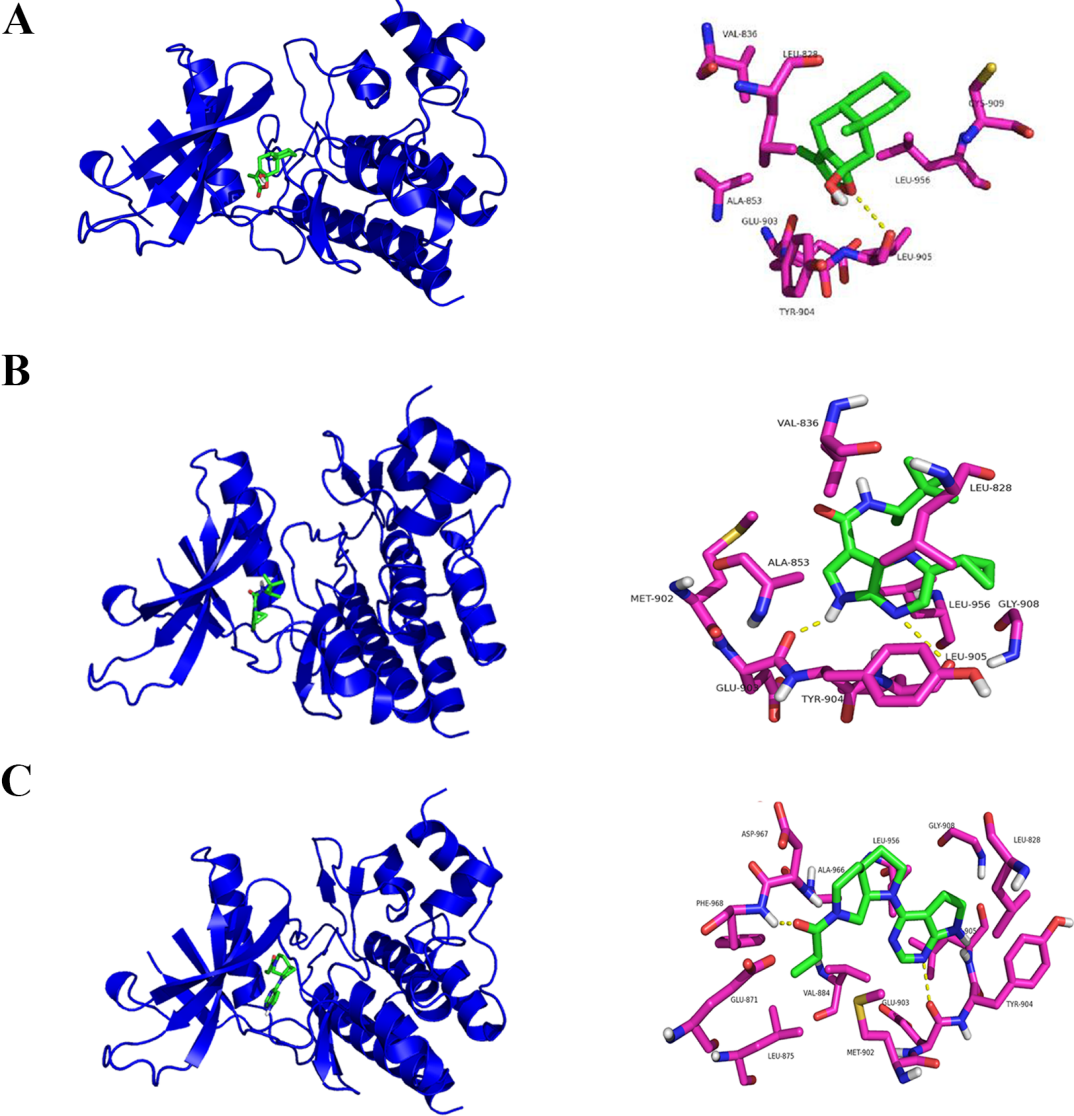
Molecular docking assay predicts atractylenolide III (ATLIII) binding closely to the Leu905 site of Jak3 protein. **(A)** Interaction diagrams of the protein Jak3 and ATLIII. Spatial location schematic diagram of the protein Jak3 and ATLIII. **(B)** Interaction diagrams of the protein Jak3 and Jak3 inhibitor 19R. Spatial location schematic diagram of the protein Jak3 and Jak3 inhibitor 19R. **(C)** Interaction diagrams of the protein Jak3 and Jak3 inhibitor 7KV. Spatial location schematic diagram of the protein Jak3 and Jak3 inhibitor 7KV.

**Figure 5:**
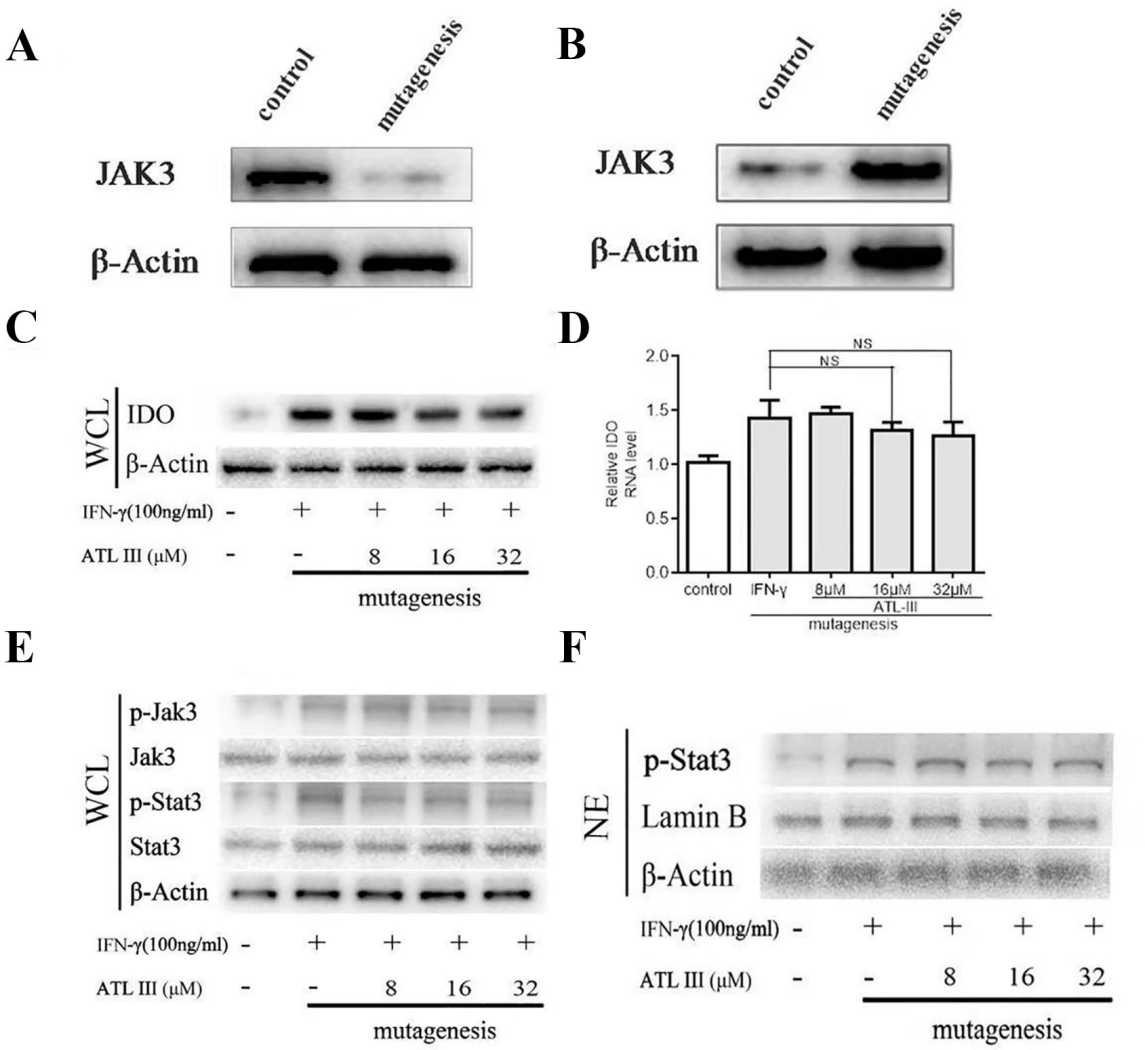
Atractylenolide III (ATLIII) fails to inhibit indoleamin-2,3-dioxygenase-1 (IDO) and the Jak3/Stat3 signaling pathway activation under forced Jak3–Leu905 mutant expression condition. **(A)** Western blotting was used to detect Jak3 protein after using CRISPR-Cas9 technology knockout. **(B)** Western blotting was used to detect Jak3 protein after transferring the Jak3–Leu905 mutant plasmid into the lung cancer cells. **(C)** Western blotting was used to detect IDO in LLC cells treated without (or with) IFN-γ (100 ng/ml) and 8, 16, or 32 μmol/l ATLIII after transfection with the Jak3 protein–Leu905 mutant plasmid. β-actin was used as an internal control. **(D)** RT-PCR detection of IDO expression in LLC cells treated with IFN-γ (100 ng/ml) or ATLIII (8, 16, or 32 μmol/l). U6 was used as an internal control. The data represent the average of three independent experiments (mean ± standard deviation). **(E)** Western blotting was used to detect Jak3, p-Jak3, Stat3, and p-Stat3 in LLC cells treated without (or with) IFN-γ (100 ng/ml) and 8, 16, or 32 μmol/l ATLIII after transfection with the Jak3 protein–Leu905 mutant plasmid in the whole cell suspension. β-actin was used as an internal control. **(F)** Western blotting was used to detect p-Stat3 in LLC cells treated without (or with) IFN-γ (100 ng/ml) and 8, 16, or 32 μmol/l ATLIII after transfection with the Jak3 protein–Leu905 mutant plasmid in the cell nucleus. β-actin was used as an internal control.

**Figure 6:**
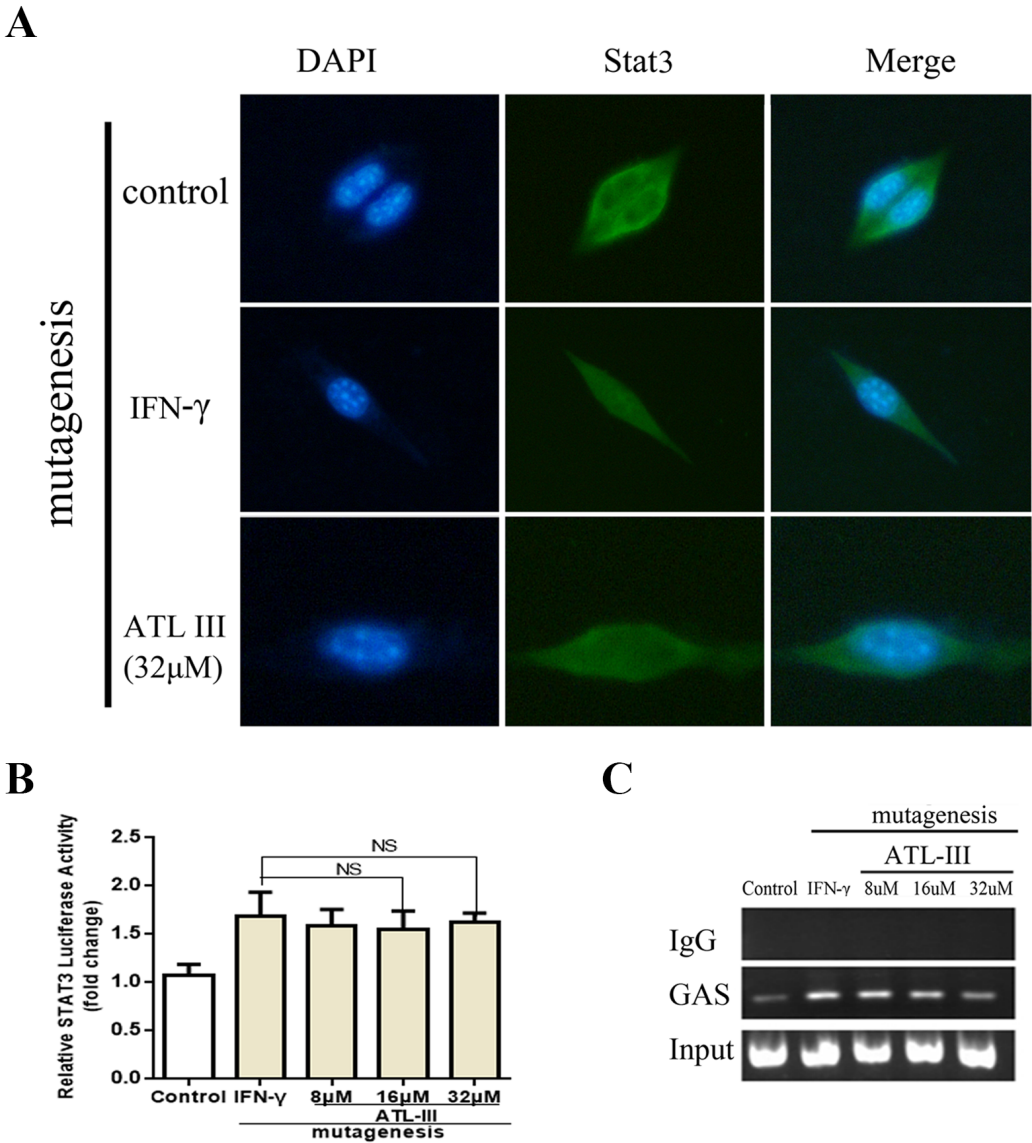
Atractylenolide III (ATLIII) loses the suppressive activity on IFN-γ-triggered Stat3 nuclear translocation and transcriptional activity under forced Jak3–Leu905 mutant expression condition. **(A)** Immunofluorescence staining detects Stat3 nuclear translocation with the Jak3 protein Leu905 site-directed mutagenesis. **(B)** Luciferase assay detects the transcriptional activity of Stat3 with the Jak3 protein Leu905 site-directed mutagenesis. The data represent the average of three independent experiments (mean ± standard deviation). **(C)** Chromatin immunoprecipitation (CHIP) assay was used to detect binding of the Stat3 and GAS promoters with the Jak3 protein Leu905 site-directed mutagenesis.

In a molecular docking experiment, we found that ATLIII entered the Jak3 target protein's active site mainly through hydrophobic and van der Waals forces. It mainly interacted with the amino acid residue of glutamic 903, leucine 905, leucine 828, valine 836, alanine 853, tyrosine 904, and leucine 956 (**Figure 4A**). When we conducted molecular docking experiments using two Jak3 inhibitors, 19R and 7KV, we found that the two residues of glutamate 903 and leucine 905 were the most likely to form hydrogen bonds with ligands. The binding conformation of ATLIII and Jak3 protein was simulated by molecular docking; the binding energy was −7.02, which acted on the active site of Jak3 in a non-covalent manner. The oxygen atom on the five-member ligand ring of ligand can form a hydrogen bond to leucine 905-NH2 with a bond length of 3 A. The formation of the hydrogen bond enhanced the affinity of ATLIII to Jak3. In comparison to the Jak3 inhibitors 19R and 7KV, we found ATLIII acting on the hydrophobic active sites. According to the docking energy analysis, the docked energy of Jak3 inhibitor 7KV was −7.36, that of 19R was −6.33, and the docked energy of ATLIII was −7.02 in between (**Figures 4A–C**). ATLIII shows no effect on Jak3/Stat3 signaling pathway activation in the forced expression of Jak3–Leu905 mutant form. We knocked out the Jak3 sequence using CRISPR-Cas9 technology and then transferred the Jak3–Leu905 mutant plasmid into the lung cancer cells. The modifications were confirmed by Western blots (**Figures 5A, B**). The luciferase reporter gene assay showed that the luciferase activity of Stat3 was significantly increased in the 100-ng/ml IFN-γ group compared with the control group. ATLIII treatment at all indicated dosages showed comparable effects with controls (**Figure 6B**), indicating ATLIII does not play a role on Stat3 inhibition in forced Jak3–Leu905 mutant expression condition. In addition, ATLIII had comparable effects on IFN-γ-induced phosphorylation of Jak3 and Stat3 in forced Jak3–Leu905 expression condition (**Figures 5E, F**). ATLIII also had no effect on IFN-γ-upregulated IDO protein level (**Figures 5C, D**). The results from immunofluorescence staining showed that Stat3 was distributed throughout the whole cell, and IFN-γ stimulation resulted in the shifting of Stat3 protein from cytoplasm to nucleus in forced Jak3–Leu905 expressed cells. ATLIII treatment showed no effect on IFN-γ stimulation-induced changes of Stat3 localization (**Figure 6A**). Furthermore, CHIP assay confirmed that Stat3 did not bind to the IDO promoter region GAS in lung cancer cells in forced Jak3–Leu905 expressed cells (**Figure 6C**). Meanwhile, the luciferase reporter gene assay showed that the transcriptional activity of Stat3 was not affected by ATLIII after transfection in forced Jak3–Leu905 expressed cells (**Figure 6B**).

### ATLIII Inhibits Interferon-Induced IDO Expression *In Vivo*

**Figure 7:**
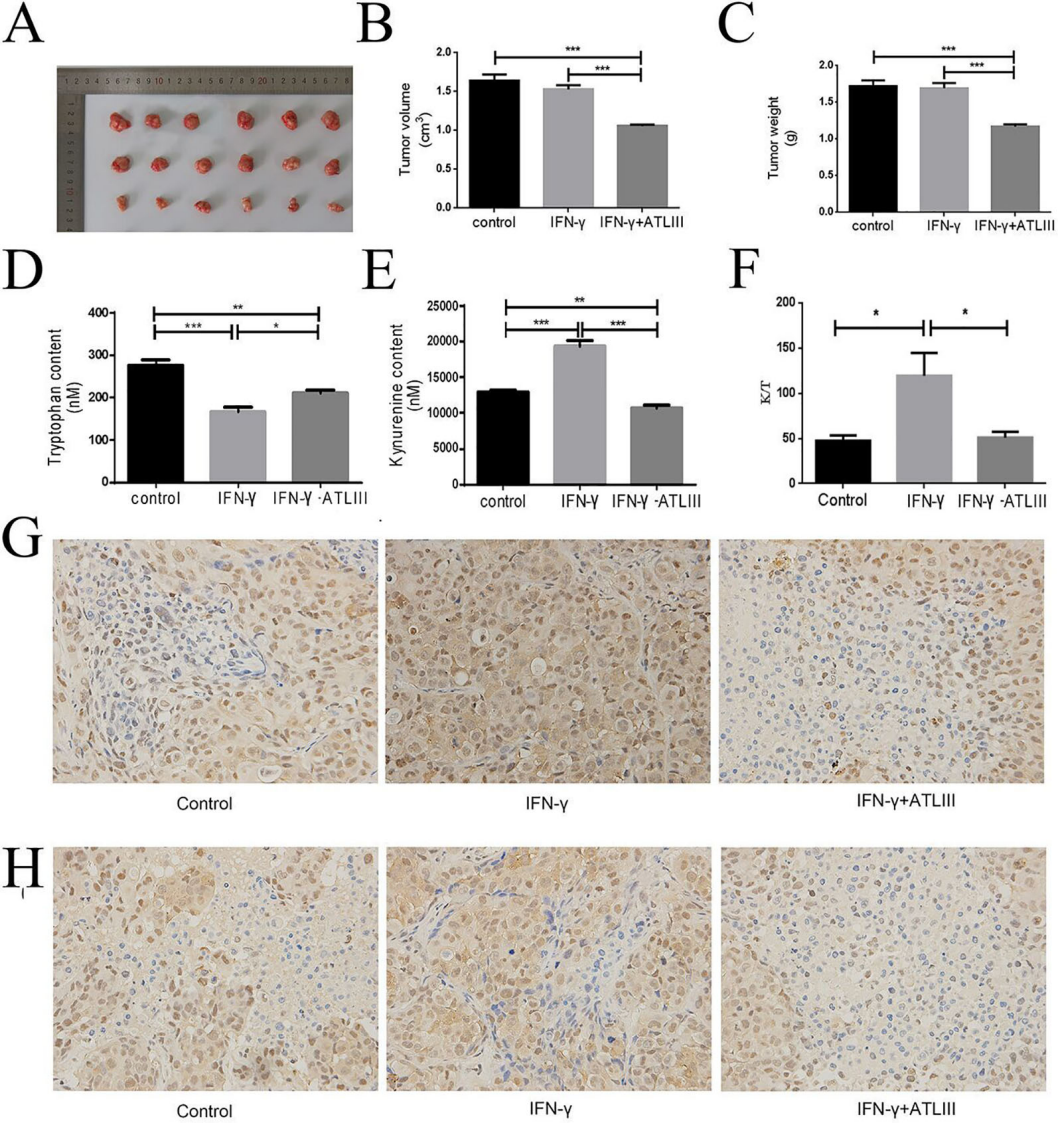
Atractylenolide III (ATLIII) inhibits tumorigenesis of lung cancer cells *in vivo*. **(A**–**C)** Tumor volume and tumor weight in the control group, IFN-γ group, and IFN-γ plus 100 mg/kg ATLIII group. **(D**–**E)** Concentrations of tryptophan and kynurenine in tumor tissues of each group by high-performance liquid chromatography (HPLC) analysis. **(F)** Expression of K/T viability assay in tumor tissues. *p < 0.05, **p < 0.01, ***p < 0.001. **(G)** Expression of p-Jak3 protein in the control group, IFN-γ group, and IFN-γ plus ATLIII group in an intravenous injection mouse model of lung cancer. **(H)** Expression of p-Stat3 protein in the control group, IFN-γ group, and IFN-γ plus ATLIII group in an intravenous injection mouse model of lung cancer. *p < 0.05, **p < 0.01, ***p < 0.001.

Then the mice experiment indicated that the tumor volume and tumor weight of the IFN-γ group had little change compared with the control group. However, the tumor volume and tumor weight were significantly decreased in the IFN-γ plus ATLIII group (**Figures 7A–C**). The experimental results showed that tryptophan was significantly downregulated while kynurenine was significantly upregulated in the IFN-γ group compared with the control. But tryptophan was significantly upregulated and kynurenine was significantly downregulated in the IFN-γ plus ATLIII group (**Figures 7D, E**). The expression of K/T in the IFN-γ group was significantly upregulated compared with the control group, while the expression of K/T was significantly downregulated in the IFN-γ plus ATLIII group (**Figure 7F**). The immunohistochemistry data showed that the expressions of p-Jak3 and p-Stat3 were significantly upregulated in the IFN-γ group compared with the control group, while the expressions of p-Jak3 and p-Stat3 were significantly downregulated in the IFN-γ plus ATLIII group (**Figures 7G, H**).

## Discussion

Targeting host immune system for cancer treatment, namely immunotherapies, has become prospect in clinical reality. Modulation of the tumor microenvironment is becoming a popular research topic in the field of immunotherapy, and the focus on checkpoint blockades in cancer immunotherapy has provided novel strategies for cancer treatment. The pervious literatures showed that ATLI, ATLII, and ATLIII display potential to regulate anti-inflammatory immune responses (Wang et al., 2009). It was also reported that ATLIII inhibits mast cell proliferation, upregulates anti-inflammatory cytokines (Yoou et al., 2017), and inhibits pro-inflammatory cytokines such as IL-6, IL-8, IL-1β, and TNF-α in tumor microenvironment. ATLIII also inhibits NF-κB and MAPK pathway activation in cancer (Ji et al., 2016). ATLIII suppresses tumor necrosis factor (TNF-α) and nitric oxide (NO)-producing macrophages in tumors (Li et al., 2007). We speculate that the anticancer effects of ATLIII are not only direct killing of tumor cell but also activating of antitumor immunity in the tumor microenvironment.

The expression of IDO is one of the major factors leading to tumor immunosuppression (McNutt, 2013), which can be detected for example in uterine, cervical, and colon cancer. The expression of IDO can be induced by IFN-γ. When IFN-γ binds to its receptor, the intracellular region of the receptor is opened, tyrosine kinase Jak is autophosphorylated, and then Jak is activated. Two Jak monomers form a homodimer groove to recruit Stat3, and the activated Stat3 forms a homodimer for transporting into the nucleus, where it then binds to GAS directly, activating the expression of the IDO promoters. However, Stat3 also induces the expression of IRF-1, which binds to the ISRE-1 and ISRE-2 elements of the IDO gene regulatory region, indirectly activating the expression of IDO (Vécsei et al., 2013). IFN-γ stimulated the expression of IDO in lung cancer cells in a dose- and time-dependent manner. Many studies have shown that IDO is a key enzyme for tumors to produce immune tolerance and can be found in ovarian, colon, and liver cancers (Cai et al., 2012; Hascitha et al., 2016), but whether IDO are expressed in lung cancer cells has not been reported. Firstly, we examined whether IFN-γ can induce the expression of IDO in lung cancer cells. Lung cancer cells were induced with different doses of IFN-γ for 12 h, and then the total protein was extracted for Western blotting to detect the expression of IDO. IDO were not expressed in the absence of IFN-γ stimulation. When the concentration of IFN-γ stimulation reached 100 ng/ml for 12 h, lung cancer cells were significantly induced to express IDO, and the expression levels of IDO increased with the increase of IFN-γ concentration. Therefore, IFN-γ stimulated the expression of IDO in a dose-dependent manner. Secondly, we examined the changes in the expression of the IDO downstream signaling molecules Jak3, p-Jak3, Stat3, and p-Stat3 induced by IFN-γ. The results showed that ATLIII can significantly inhibit the phosphorylation of Jak3 and Stat3 and the nuclear translocation of Stat3, which significantly inhibited the activation of the IDO gene promoters, thereby inhibiting the expression of IDO. Molecular docking experiments were used to predict the binding site of ATLIII to Jak3 protein and found that it binds to the Jak3 leucine 905 site. We then found that ATLIII does not regulate the expression of IDO in a Jak3 leucine 905 mutant. Based on the above results, we speculate that the molecular mechanism of ATLIII may be through inhibiting the phosphorylation of Jak3 and Stat3 and the nuclear translocation of Stat3, thus affecting the expression of IDO by IFN-γ and regulated the immune microenvironment. Moreover, the interaction site of ATLIII with the Jak3 protein was leucine 905 *via* a hydrogen bond. After transfection with the Jak3 protein–Leu905 mutant plasmid, ATLIII can no longer play a role. This study elucidated a new mechanism for the anticancer effect of ATLIII, which may provide a feasible target for the clinical immunotherapy of malignant tumors.

## Data Availability Statement

All datasets generated for this study are included in the article/supplementary material.

## Ethics Statement

The animal study was reviewed and approved by the Experimental Animal Ethics Committee of Nanjing Medical University.

## Author Contributions

CY designed all the experiments. J-BL did the cell experiment. DC did the animal experiment. T-TB wrote the article. F-TF conducted the animal experiment.

## Funding

This study was supported by the China Postdoctoral Science Foundation (2018M632267) and the talents program of Jiangsu Cancer Hospital (YC201815). 5451 Project of Health Ministry in Henan Province (No 201177).

## Conflict of Interest

The authors declare that the research was conducted in the absence of any commercial or financial relationships that could be construed as a potential conflict of interest.
